# Multidrug Resistance in Mammals and Fungi—From MDR to PDR: A Rocky Road from Atomic Structures to Transport Mechanisms

**DOI:** 10.3390/ijms22094806

**Published:** 2021-04-30

**Authors:** Narakorn Khunweeraphong, Karl Kuchler

**Affiliations:** Center for Medical Biochemistry, Max Perutz Labs Vienna, Campus Vienna Biocenter, Medical University of Vienna, Dr. Bohr-Gasse 9/2, A-1030 Vienna, Austria; narakorn.khunweeraphong@meduniwien.ac.at

**Keywords:** yeast, multidrug transporter, anticancer, antifungal resistance, ABC transporters, mechanism

## Abstract

Multidrug resistance (MDR) can be a serious complication for the treatment of cancer as well as for microbial and parasitic infections. Dysregulated overexpression of several members of the ATP-binding cassette transporter families have been intimately linked to MDR phenomena. Three paradigm ABC transporter members, ABCB1 (P-gp), ABCC1 (MRP1) and ABCG2 (BCRP) appear to act as brothers in arms in promoting or causing MDR in a variety of therapeutic cancer settings. However, their molecular mechanisms of action, the basis for their broad and overlapping substrate selectivity, remains ill-posed. The rapidly increasing numbers of high-resolution atomic structures from X-ray crystallography or cryo-EM of mammalian ABC multidrug transporters initiated a new era towards a better understanding of structure–function relationships, and for the dynamics and mechanisms driving their transport cycles. In addition, the atomic structures offered new evolutionary perspectives in cases where transport systems have been structurally conserved from bacteria to humans, including the pleiotropic drug resistance (PDR) family in fungal pathogens for which high resolution structures are as yet unavailable. In this review, we will focus the discussion on comparative mechanisms of mammalian ABCG and fungal PDR transporters, owing to their close evolutionary relationships. In fact, the atomic structures of ABCG2 offer excellent models for a better understanding of fungal PDR transporters. Based on comparative structural models of ABCG transporters and fungal PDRs, we propose closely related or even conserved catalytic cycles, thus offering new therapeutic perspectives for preventing MDR in infectious disease settings.

## 1. Introduction

### 1.1. ABC Transporters and Clinical Relevance of MDR

The ATP-binding cassette (ABC) transporter family is one of the largest protein superfamilies present in all living organisms, from prokaryotes to eukaryotes [1,2,3,4,5]. ABC transporters can operate as exporters or importers in an ATP-dependent manner, and mediate the membrane translocation of bewildering substrate spectra against concentration gradients [6,7,8]. In addition, ABC proteins can function as ion channels, channel regulators, receptors, proteases, protein sensors or are even involved in mRNA translation and ribosome biogenesis [9,10,11]. Remarkably, conserved architectures offer specific yet broad substrate-binding regions and somehow form a translocation path that operates in a unidirectional way in eukaryotes. The wide substrate range includes cationic anticancer drugs, antifungal drugs, steroids, phospholipids, bile acids, antibiotics, peptides, ions, heavy metals, carbohydrates and glucocorticoids, as well as toxins [12,13,14,15]. The hallmark domain organization of ABC transporters entails four core units, two evolutionarily conserved nucleotide-binding domains (NBDs) and two transmembrane-spanning domains (TMDs), typically consisting of twelve hydrophobic transmembrane-spanning helices (TMHs). These four domains are normally arranged as a full-transporter in a single protein as TMD1–NBD1–TMD2–NBD2 or in a reverse configuration NBD1–TMD1–NBD2–TMD2. Alternatively, half-transporters come in NBD–TMD or TMD–NBD arrangements, which require at least homo- or hetero-dimerization for a functional complex [16,17,18,19]. In addition, some members contain additional domains or motifs such as the TMD0 domain or the R-domain regulatory motif [8,20,21].

The NBD is a universally conserved domain that consumes ATP and somehow fuels the dynamic switch of the transporter structure from an inward substrate-binding state to an outward substrate-releasing conformation. The catalytic cycle drives the conformational switch at the TMD and enables the substrate translocation through an as yet elusive transport pathway [22,23]. The TMDs are more diverse in sequence and show much less conservation, but clearly are essential for forming putative substrate translocation pores. They must handle a broad spectrum of chemically diverse substrates and inhibitors [24,25]. Notably, the communication between NBD and TMD and the dynamics underlying the entire transport cycle of ABC transporters remain unclear. While certain elements or stages of the transport cycles may be conserved among subfamilies, the expanding number of atomic structures and the resulting mechanistic information for distinct ABC transporters make unifying mechanisms less likely, challenging earlier notions about a unified catalytic cycle [8,16,17,26,27,28,29,30,31,32,33,34,35,36,37,38,39,40,41,42,43,44,45]. No matter what the actual catalytic cycle or mechanism of a given type I or type II exporter may be, a tantalizing possibility is that a basic conserved mechanism operates for PDR and ABCG, but slightly different transport mechanisms could be a consequence of the nature of substrates that would distinctly affect the kinetics and dynamics of the cycle.

The human ABC transporter family of 48 genes served to categorize subfamilies into the ABCA to ABCG nomenclature [46,47,48], although the surge of recent atomic structures and functional considerations made it clear that a new and improved nomenclature based on structure–function relationships is needed [43]. Remarkably, inborn errors of several human ABC transporters lead to prominent genetic diseases [49], including cystic fibrosis (ABCC7 or CFTR) [50,51], hepatic cholestasis (ABCB11 or BSEP) [52,53], plant sterol sitosterolemia (ABCG5/G8) [54,55,56,57], neonatal hyperinsulinemic hypoglycemia or non-insulin-dependent childhood diabetes (ABCC8) [58], gout (ABCG2 or BCRP) [59], Dubin–Johnson syndrome (ABCC2 or MRP2) [60,61] and Stargardt’s macular dystrophies and retinophathies (ABCA4) [62], peroxisomal adrenoleukodystrophy or ALD (ABCD1) [63,64,65], immune deficiency—class I MHC antigen presentation (ABCB2/B3, TAP) [66,67,68], cholesterol transport and HDL assembly or Tangier’s disease (ABCA1, ABCG1) [69,70,71,72,73,74,75], pseudoxanthoma elasticum or PXE (ABCC6) [76,77,78,79,80,81,82], dilated cardiomyopathy (ABCC9) [83], defective earwax synthesis (ABCC11) [84,85], lung surfactant deficiency (ABCA3) [86,87,88,89], lamella and harlequin ichthyosis (ABCA12) [90,91,92], pregnancy-related cholestasis (ABCB4) [93,94] and sideroblastic anemia (ABCB7) [95,96,97,98].

### 1.2. Mammalian ABC Multidrug Transporters

Most, if not all, eukaryotic ABC transporters function as unidirectional exporters and use ATP consumption to drive transport. Some have been implicated in uptake processes as well, although this remains controversial [39,99]. Importantly, ectopic or dysregulated overexpression of certain ABC transporters often contributes to or promotes MDR phenomena in several but not all human cancer types [2,10,11,100,101,102]. Based on sequence similarity and domain arrangement, mammalian ABCs fall into two major groups, referred to as type I and type II exporters [12], although recently, a new classification has been proposed [18]. At least three MDR exporters have been linked to MDR in human tumors, including P-glycoprotein (P-gp/MDR1/ABCB1) [103,104,105,106], MRP1 (MDR-associated protein 1/ABCC1) [107] and BCRP (Breast Cancer Resistance/ABCG2) [108,109,110]. All three share rather broad and partially overlapping drug specificity [14,111]. Most substrates of ABCB1 and ABCG2 are cationic hydrophobic compounds [112], which may probably be expelled directly from the lipid phase as originally proposed by the “hydrophobic vacuum cleaner” model [105,113,114] or from the outer membrane leaflet by a floppase-like function [5,14,19,109,111,115,116]. A similar mechanism has been proposed in ABCA1 for the cholesterol loading of apolipoprotein A-I (apoA-I) [5,116,117]. P-gp, MRP1 and ABCG2 are normally residing in the plasma membrane of epithelial organ linings (such as liver, intestine, blood–brain barrier, placenta and mammary epithelium) [118,119]. Their physiological tasks include vital roles in cellular detoxification and in organ protection by excretion of toxic compounds or xenobiotic molecules [120]. Substrates are amphipathic, lipid-soluble compounds of extremely diverse chemical spaces, ranging from small molecules to bulky lipophilic cations and conjugated organic anions [14,112,121]. 

ABCB1 or P-glycoprotein (P-gp) or MDR1 (encoded by the *MDR1* gene) was identified from a multidrug-resistant KB carcinoma cell line [103,122,123] as the first mammalian type I exporter class. P-gp is expressed on apical membranes of epithelial cells in colon, small intestine, liver, placenta, kidney, gut, pancreatic, bile duct and blood–brain barrier [124,125]. Homozygous P-gp knock-out mice showed a 100-fold increase in drug (ivormectin) permeability at the blood–brain barrier, which led to the discovery of its physiological role in organ protection [126,127,128]. ABCB1 transports a diverse array of substances, including chemotherapeutic drugs, steroids, several phospholipids, fluorescent dyes, peptides and ionophores [113]. Furthermore, ABCB1 is believed to function as a floppase-like lipid transporter [43,129]. Despite huge therapeutic promises, numerous clinical studies on ABCB1 inhibitors or reversal agents [130,131,132,133,134,135,136,137,138] showed marginal to no benefits, thus rendering attempts to translate P-gp inhibitors into the clinic so far futile efforts [111,139]. Of note, despite an almost highly conserved primary sequence identity, the closest P-gp homologue, ABCB4/MDR2, has not been implicated in drug transport or cancer MDR [140], as it resides in the canalicular and appears to have a restricted substrate spectrum limited to phosphatidycholine-related phospholipids in the canalicular membrane.

ABCC1 or MRP1 or multidrug resistance-associated protein1 (encoded by the *ABCC1* gene) was discovered as the second member of MDR exporters, cloned from a multidrug-resistant P-gp-negative human lung cancer cell line with doxorubicin tolerance [107]. ABCC1 is mostly on the basolateral surface of polarized epithelial cells, with moderate to high abundance in the gastrointestinal tract, kidney, bladder, testis, ovary, endometrium, adipose tissues, appendix and tonsils. Low-level expression is found in brain, lung, liver, gall bladder, pancreas, bone marrow and skin [141,142,143] to excrete a variety of endogenous substances, including glutathione, prostaglandins, C4-leukotrienes glucuronide conjugates, sulfate conjugates, heavy metal oxyanions and, most importantly, conjugated metabolites of otherwise hydrophobic compounds [14,112,144,145]. 

ABCG2 or BCRP or Breast Cancer Resistance Protein (encoded by the *ABCG2* gene) was originally isolated from P-gp-negative multidrug-resistance breast cancer cell lines [109,110]. ABCG2 homes to the apical membranes in many epithelial cells and tissues, including lung, gut, intestine, liver, breast, placenta, hematopoietic stem cells and especially in the blood–brain barrier [109,110,119,146]. ABCG2 is a half-transporter carrying a TMD at the C-terminus, requiring homo-dimerization to form a full functional molecule. ABCG2 is overexpressed in many solid tumors as well as acute myeloid leukemia (AML) and acute lymphocytic leukemia (ALL). Dysregulated ABCG2 overexpression is linked with poor prognosis in several cancer types [139,147], with particularly low survival in AML patients [134,137,148,149,150,151]. Like P-gp, ABCG2, as well as PDRs such as Yor1, Pdr5, Cdr1 or Snq2, show extremely broad substrate specificity (Table 1), all in all transporting hundreds of diverse compounds, including dietary xenobiotics, toxins, metabolites, vitamins, lipids, steroids, antibiotics and antifungal as well as anticancer drugs [152,153,154,155]. Of note, the many exceptions seen for each transporter make a generalization of substrate preferences for a given ABCG or PDR transporter challenging without experimental evidence. Remarkably, however, despite their pronounced structural conservation, additional ABCG family members such as ABCG1, ABCG4 and the heterodimeric ABCG5/G8 transporter have not been associated with MDR phenotypes in cancer [15,115,156,157,158,159,160,161,162]. 

Collectively, P-gp, MRP1 and ABCG2 act as brothers in arms to ensure the physiological detoxification of endogenous metabolites as well as exogenous xenobiotics across most epithelial barriers, including placenta, testis, mammary epithelium, liver and GI tract as well as the blood–brain barrier [119,139,146,152,206]. However, how, and sometimes even if, they actually cause clinical MDR in cancer has remained a highly controversial issue, often subject to intense discussions in the field [102,207]. As for microbial anti-infective MDR, it has been generally accepted though that bacterial ABC transporters [208,209,210,211,212] and fungal PDR transporters [124,213,214,215,216] are key causes for clinical MDR, often setting an unsurmountable roadblock in antimicrobial treatments [10,101,217,218,219].

### 1.3. ABC Multidrug Transporters in Fungal Kingdoms

Invasive fungal diseases account for ~1.5 million deaths per year worldwide [220,221]. The increasing numbers of immunosuppressed people, including the elderly, transplant recipients, cancer and HIV/AIDS patients will most likely increase future cases of infections by opportunistic pathogens like *Candida* species (spp.) [222]. Candida spp. are commensal colonizers and part of the microflora present on mucosal and epithelial barriers such as the gastrointestinal and urogenital tracts. *Candida* spp. are a major part of the physiological mycobiome species [223], along with several thousand bacterial species constituting tissue-specific microbiomes [224,225,226,227,228,229]. Several *Candida* spp. can cause life-threatening invasive systemic disease in severely immunocompromised individuals [230]. The small number of chemical entities in antifungal drugs have been very problematic in the past, especially after prolonged use or after extensive prophylaxis, as was the case for pronounced azole resistance in HIV patients. Hence, the propensity to develop MDR is lower when larger drug arsenals against different targets are available [231]. For example, *C. auris* is a newly emerging pan-resistant fungal pathogen first reported from an ear infection in Japan in 2009 [232]. Within a decade, *C. auris* appeared in more than 40 countries around the globe, causing hospital outbreaks of invasive candidemia [233,234,235,236,237,238,239,240]. Importantly, *C. auris* is also causing severe superinfections with viruses, as seen in a recent co-infection of COVID-19 patients in a Mexican hospital ICU, leading to a dramatic overall mortality of 83%. In fact, intrinsic MDR in other *Candida* spp. such as *C. glabrata* and *C. kruzei* have also been increasing over the last decade [230,241,242]. Importantly, among other mechanisms, such as drug target gene mutations [243,244], efflux-based MDR/PDR has been recognized as a major cause of fungal anti-infective drug resistance [219,235,244,245,246,247]. 

**Figure 1:**
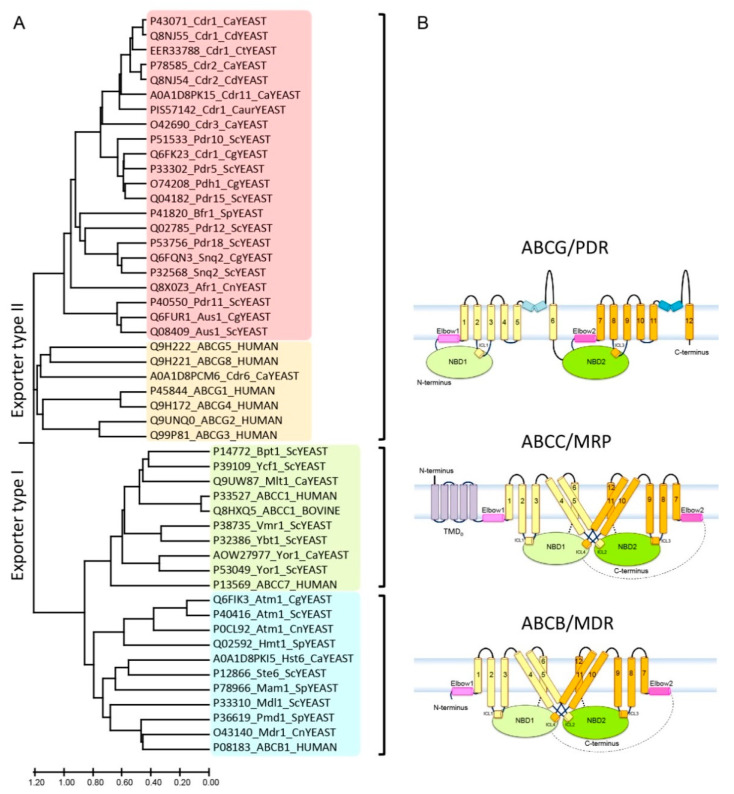
Phylogeny and structural organization of ABC transporters in mammals and yeast. (**A**) The phylogenetic tree shows the evolutionary relationships of ABC transporter subfamilies in yeast and mammals. Some 29 ABC transporters were subjected to amino acid sequence alignments. Branch length was analyzed using MEGA-X, and represents the evolutionary distance in the units of the number of amino acid substitutions per site. Names are given in the UniProt code, protein name and organism, respectively. The analysis reveals two major exporters subfamilies referred to as type I and II. Type I was sub-classified into ABCB/MDR (blue) and ABCC/MRP (green) subgroups. The type II family represents ABCG/PDR subgroups with the fungal (red) and mammalian transporters (orange). Ca: *Candida albicans*, Cg: *Candida glabrata*, Caur: *Candida auris*, Cd: *Candida dubliniensis*, Ct: *Candida tropicalis*, Cn: *Cryptococcus neoformans*, Sc: *Saccharomyces cerevisiae*, Sp: *Schizosaccharomyces pombe*. (**B**) Predicted membrane topologies of three MDR ABC exporter families. The transporters hold several diagnostic hallmark domains, including two NBDs (NBD1: light green; NBD2: green), two TMD regions usually with 6 putative membrane-spanning helices each (TMD1: light yellow; TMD2: bright orange), elbow helix (pink), re-entry helix (blue) and TMD0 (purple), respectively.

Fungal ABC proteins are also quite diverse and are implicated in many biological functions, contributing to pivotal cellular processes, including cellular detoxification and stress adaptation [214,248,249]. Owing to space constraints, we will limit our discussion to paradigm fungal PDR family members implicated in drug resistance phenomena, but refer the reader to numerous recent reviews discussing fungal ABC proteins at large [249,250]. Fungal ABC proteins of the PDR subfamily are the closest eukaryotic orthologues of human ABCG family exporters [245,251]. For instance, *C. albicans* and *C. glabrata* harbor 27 and 18 ABC proteins in total, respectively [250]. Interestingly, *Cryptococcus neoformans* and *Aspergillus fumigatus* harbor even larger numbers, with 54 and 49 ABC transporters, respectively [252,253]. While we are not discussing transporters which are not linked to MDR phenomena, we still provide a comprehensive list of all known ABC transporters in non-pathogenic yeasts (Table 1) and their phylogenetic relationships (Figure 1A). MRP-like yeast ABC transporters including Yor1, Ycf1, Ybt1, Vmr1 and Bpt1 mediate vacuolar detoxification and heavy metal resistance [214]. Of note, Ycf1, which also has a rudimentary R-domain motif as present in human CFTR [50], was the first yeast homologue of mammalian MRP [254], while pathogenic fungi such as *C. albicans* harbor only Yor1 and Mlt1 (Table 2).

The fungal PDR (ABCG) or pleiotropic drug resistance family is the largest subfamily of ABC transporters of the type II exporter class [251]. PDR transporters are the closest structural orthologues of all mammalian ABCG subfamily transporters (Figure 1A), sharing the same topological orientation and domain arrangements with mammalian ABCG5/ABCG8 [255] and ABCG2 [256,257,258,259] (Supplementary Figure S1). PDR transporters in fungal pathogens implicated in clinical antifungal resistance in *C. albicans* include Cdr1, Cdr2, Cdr3, Cdr6 and Cdr11, in *C. glabrata* (Cdr1, Pdh1, Snq2 and Aus1) [260], in *C. auris* (Cdr1), in *C. tropicalis* (Cdr1), in *C. dubliniensis* (Cdr1 and Cdr2) and in *Cryptococcus neoformans* (Afr1) (Table 2). All are highly conserved in non-pathogenic yeasts (Table 1). Their overexpression in pathogens causes hallmark MDR phenotypes seen for mammalian or bacterial ABC transporters [111,141,217,218,235,261,262,263,264]. 

Diversity of fungal PDR transporters and evolutionary relationships to mammalian ABC transporters. Most ABC proteins from non-pathogenic baker’s yeasts (Table 1) are also found in various numbers and functions in pathogenic fungi (Table 2). A phylogenetic tree analysis suggests two major groups, referred to as exporter type I and type II [4,248,265,266], contain three MDR, MRP and PDR subfamilies (Figure 1A). While type I exporters (MDR/ABCB and MRP/ABCC) hold a TMD1­–NBD1–TMD2–NBD2 configuration, all PDR/ABCG type II exporters adopt a “reverse” architecture, such as NBD1–TMD1–NBD2–TMD2 (Figure 1B). By contrast with the mammalian ABCG subfamily, all fungal PDR proteins are full-size transporters (Figure 1B). 

## 2. Atomic Structures of Eukaryotic Multidrug ABC Efflux Exporters

**Figure 2:**
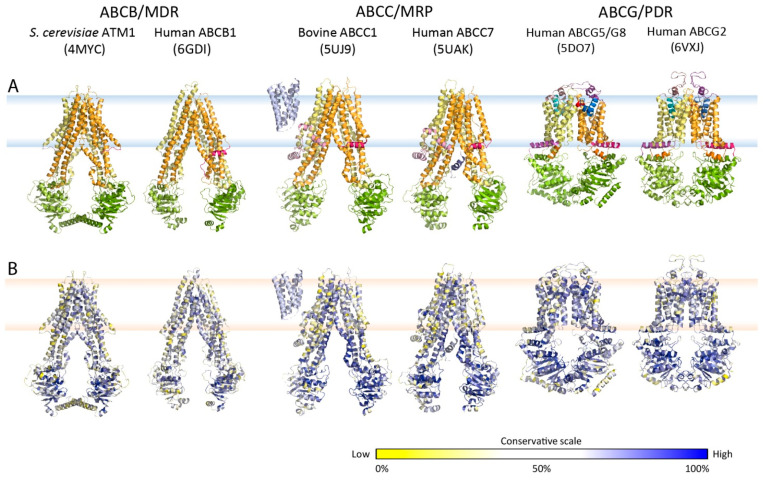
Atomic structures, folds and evolutionary conservation of MDR exporters. (**A**) Crystal or cryo-EM particle structures of multidrug resistance-related ABC exporters from the type I (ABCB and ABCC) and type II families (ABCG subfamily) using the color codes as shown in Figure 1B. (**B**) Conservation analysis of ABC exporters from panel A. Multiple sequence alignments of amino acid sequences from Table 1 and Table 2 were generated for all subfamilies. The degree of conservation was calculated and indicated as a color gradient ranging from low conservation (yellow) to high conservation (blue).

Several high-resolution structures, obtained through X-ray crystallography or cryo-EM approaches in the past five years, provided a better understanding of the structure–function relationships of mammalian type I and type II exporters (Table 3). However, despite ever-increasing structural information, the path from static atomic structures to precise molecular mechanisms has turned out to be a rocky road for scientists and drug discovery. Thus, we are still far from understanding the conformational dynamics as well as the mechanics driving their transport cycles. Each half-molecule of ABCB and ABCC type I exporters harbor at least six transmembrane helices that extend into the intracellular loops linked to their coupling helices, thus connecting to both the proximal NBD and crossing over to the distal NBD [8,31,39,293,294,295,296]. In the ATP-free apo state, if it ever exists inside cells, these exporters maintain an inward-facing configuration, whereby both NBDs appear apart to offer access for ATP and possibly substrates (Figure 2A) [297]. Importantly, while the ABCC subfamily shares a highly similar overall fold with ABCB, MRP transporters have an additional N-terminal domain known as TMD0 [20,298,299]. Notably, CFTR also adds the so-called R(egulatory)-domain residing in the core of the channel between the N-terminal and the C-terminal TMDs [21,300,301] (Figure 1B).

At present, the available atomic structures of type I exporters are as listed in Table 3. For the ABCB1 subfamily, many conformations, including inward-facing, outward-facing, occluded state, substrate-bound, inhibitor-bound and antibody-bound, have been solved, all in all yielding 24 structures in the PDB [296,302,303,304,305,306,307,308,309,310]. For the ABCC subfamily, four cryo-EM structures are available for bovine ABCC1 in the PDB [311,312]. Furthermore, seven structures are available for CFTR [300,301,313,314,315], and 12 structures for ABCC8 or SUR1 [316,317,318,319,320]. Strikingly, the first atomic structure of the heterodimeric ABCG subfamily member ABCG5/G8 came as a surprise, as the X-ray crystals revealed an unexpected compact fold, in which NBDs were located in close proximity to the TMDs. In addition, the molecule held a lid-like structure at the extracellular roof of the translocation pathway. This fold resembled bacterial importers more than a prototypic export pump [38]. This structure paved the way for solving the human ABCG2 transporter, for which now 11 atomic structures are available in the PDB [256,257,258,259] (Table 3).

## 3. Key Residues and Motifs Are Conserved in Multidrug Transporters ABCG/PDR

The mammalian ABCGs are the closest orthologues of yeast PDR transporters [216,245,250,251,321], especially Pdr5 and Cdr1 [166,322,323] (Supplementary Figure S1). To identify conserved regions of fungal ABC proteins based on mammalian ABC structures, we determined a conservation score for the fungal PDR family, and mapped conserved residues into the atomic structures of mammalian orthologues (Figure 2B). We subjected the fungal ABCG/PDR subfamily to multiple sequence alignments with the mammalian ABCGs (Supplementary Figure S1), showing that NBDs hold several highly conserved motifs related to ATP consumption. Although TMDs are usually more diverse, several regions are also preserved between PDR and ABCG, implying that these domains are pivotal for an evolutionarily related catalytic cycle.

Conservation in the NBD. First, the NBDs in fungal PDRs share highly conserved motifs as well as residues with ABCG required for ATP-binding and hydrolysis [45,153,324,325,326], including Walker A, Q-loop, Hot spot helix, Signature motif, Pro-loop, Walker B, D-loop and H-loop, respectively [327]. The alignment indicates that fungal NBDs adopt a RecA-like structure, an ATPase-containing fold that was first seen in RecA, which is involved in DNA recombination, and was later found in many ATPases [328] (Supplementary Figure S1 and Table 4). There are only minor differences though, as ABCG2 and ABCG5/G8 are half-transporters that require homo- or hetero-dimerization, while all fungal PDRs are full-size transporters, some of which with asymmetric deviant ATP-binding sites (Table 4). In the first NBD of PDR transporters, the glutamine (Q) in the Q-loop is replaced by glutamate (E), the Pro-loop disappeared and histidine (H) in the H-loop is substituted by tyrosine (Y). Hence, the notion emerged that fungal PDRs would follow an asymmetric catalytic cycle [173,216,306,329,330].

Conservation in the TMD region. Second, the general architecture and configuration of TMDs are maintained, as each TMD in ABCG/PDR transporters contains six putative membrane-spanning helices, and a rather short first intracellular loop contains the coupling helix. The large ECL is part of the lid architecture in the extracellular region. Interestingly, two putative helices residing at both lipid bilayer leaflets, which are important for function, as they restrict dynamic movements during transport cycle, are only found in the ABCG subfamily [40,42]. The amino acid alignments reveal the consensus sites present in conserved motifs and domains (Figure 1B and Supplementary Figure S1).

The elbow helix is a 15-residue amphipathic helix sharing highly conserved residues among all ABCG/PDR members. This elbow helix is an intrinsic part of the triple helical bundle (THB), which also engages the hot spot helix from the NBD and the coupling helix from the first intracellular loop (ICL1) [332]. Importantly, the center of the elbow helix contains a highly conserved arginine (R), which is essential for a salt bridge interaction with a glutamate (E) residue in ICL1 to stabilize the THB [40].

The transmembrane helix 1 (TMH1) is a prototypic 20-residue membrane-spanning helix, following the elbow helix in all ABCG/PDR exporters. The small extracellular loop 1 (ECL1) is a short linker connecting TMH1 and TMH2 at the cell surface. Notably, ECL1 in the first half holds a conserved arginine (R), which is important for salt bridge formation with a conserved glutamate (E) in the re-entry helix of the same molecule [42]. TMH2 is thought to be a part of the substrate/inhibitor binding zone in human ABCG2 [333]. A conserved phenylalanine (F) in the middle of TMH2 may be a recognition site for both substrates and inhibitors [333].

The intracellular loop 1 (ICL1) spans over 30 residues in the ABCG/PDR subfamily and holds a critical U-turn motif. ICL1 operates as the coupling helix for the NBD–TMD communication by participating in a triple helical bundle (THB). The entire THB functions as a molecular spring that controls the catalytic cycle by regulating the conformational switch. Indeed, two conserved negative residues within ICL1 of human ABCG2, E451 and E585, are thought to control the intracellular gating mechanism and engage in salt bridges with the elbow helix, respectively. Remarkably, ICL1 holds an essential tyrosine residue that is conserved in all ABCG/PDR transporters. This Y464 contributes a salt bridge interaction within the THB complex for stabilization of the transmission interface.

TMH3 also has several conserved residues within the ABCG/PDR group such as negative residues (E or D), proline, as well as positive amino acids (K or R). D477 at the start of TMH3 in human ABCG2 is on top of the transmission interface and may contribute to an intracellular gate. ECL2 is again rather short, containing only eight residues with a kink, connecting TMH3 and TMH4 at the cell surface.

The hydrophobic valve is a kinked domain just after TMH5, consisting of the conserved “glycine-Φ-Φ” (Φ is hydrophobic residue) motif that subtends the extracellular bilayer leaflet. The valve is pivotal for controlling substrate transport through a putative translocation pathway whose precise nature and dynamics remains obscure. The re-entry helix is the counterpart of the elbow helix and is unique, establishing a kinked hallmark motif in the ABCG/PDR subfamily with several conserved residues. In addition, ECL3 is the only large extracellular loop, and a main part of the extracellular roof architecture. Interestingly, ECL3 in human ABCG2 has three cysteines, which form intra- and inter-molecular disulfide formations, and perhaps facilitate dimerization and drug release [334,335]. Likewise, conserved cysteines in fungal PDR transporters may engage in an intra-molecular disulfide bond to stabilize the PDR biogenesis. Finally, TMH6 is followed by a very small but highly conserved C-terminus that contains several positive charges at the C-terminus.

## 4. *C. albicans* PDR/Cdr1 Holds All Conserved Motifs Critical for ABCG Function

**Figure 3:**
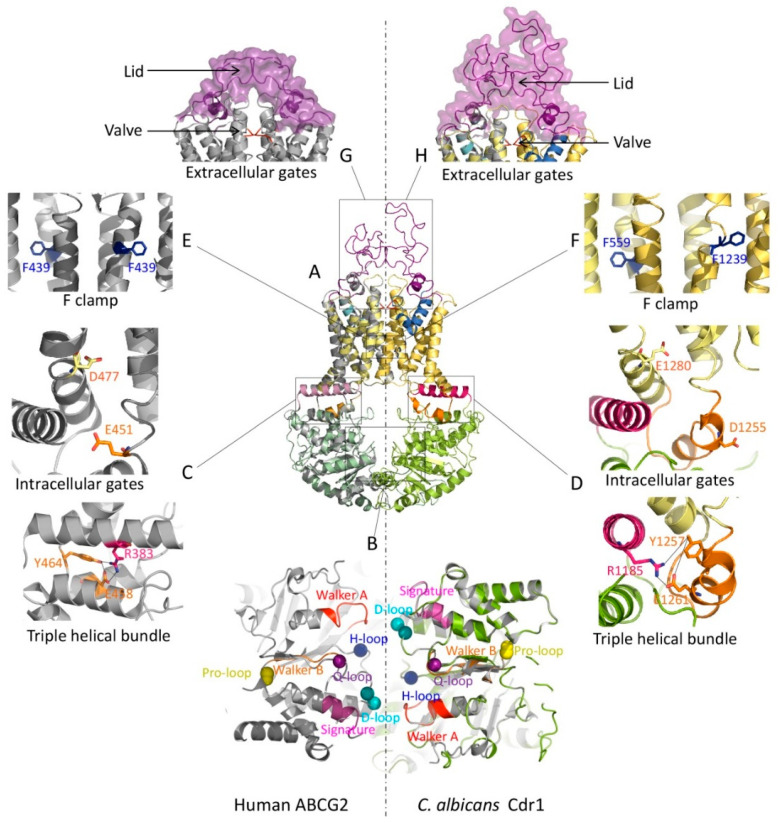
Type II mammalian and fungal ABCG/PDR exporters share functional domains. (**A**) A homology model of the PDR exporter Cdr1 from *C. albicans* was generated using the SWISS-MODEL tool (color ribbon, NBD: green, TMD: yellow, elbow helix: pink, ICL: orange, valve: red, re-entry helix: blue and ECL: purple). The Cdr1 model was superimposed with the cryo-EM structure of human ABCG2 (PDB ID: 6VXF) (gray ribbon). (**B**) Zoom-in top view of the NBD dimer from panel A at the NBD–NBD interface shows essential conserved motifs (Walker A: red, Q-loop: violet, signature: pink, Pro-loop: yellow, Walker B: orange, D-loop: cyan and H-loop: blue). The transmission interfaces of human ABCG2 (**C**) and *C. albicans* Cdr1 (**D**) show a network cluster of triple helical bundles (THB) with conserved tyrosine residues as part of a salt bridge between elbow helix and ICL. Two negative residues are shown as the intracellular gates. Conserved residues are shown as sticks with color-coding as in the topology model. The conserved phenylalanine F clamp F439 in human ABCG2 (**E**) and the putative F clamp in *C. albicans* Cdr1 (**F**) are indicated as sticks. At the extracellular gates, the valve-like structures at the top of central cavity (red) are shown at the corresponding position of human ABCG2 (**G**) and *C. albicans* Cdr1 (**H**). The lid-structure formed by ECLs is shown as a violet ribbon with a surface. The homo-dimeric human ABCG2 transporter has a symmetric lid (**G**), while the full-size *C. albicans* Cdr1 transporter has a larger ECL forming the outer lid and part of the roof architecture (**H**).

The atomic structures from X-ray crystallography and cryo-EM of mammalian ABCGs suggest a rather unique fold resembling an importer rather than an exporter [38,256,257,258,259]. Indeed, mammalian ABCGs and fungal PDR exporters share conserved and superimposable topologies with all functional motifs in equivalent places (Figure 1A, Figure 2B, Supplementary Figure S1 and Table 4). Hence, we generated homology models of Cdr1 from *C. albicans*, using human ABCG2 (PDB ID 6VXF) as a template (Figure 3A). Remarkably, the Cdr1 structural model perfectly mirrored the human ABCG2 conformation, since each pivotal motif important for function is also present in Cdr1 (Figure 3). 

The NBDs in Cdr1 are in closer contact and hold all conserved regions required for ABCG2 function (ATPase activity, A-loop, Walker A and B, Q-loop, mutational hot spot helix, signature loop, Pro-loop, D-loop and H-loop). The N-terminal and C-terminal NBDs form a head-to-tail dimer upon ATP-binding [336,337], with a RecA-like and an α-helical subdomain [327] (Figure 3B). Nonetheless, the fungal NBD1 has three minor differences, in that (i) a glutamine residue in the Q-loop is replaced with glutamate, (ii) the histidine residue in the H-loop is substituted with tyrosine and (iii) the Pro-loop is missing but contains a glycine instead [337] (Table 4). The non-identical deviant NBDs in fungal PDRs [41,216,338,339] may support an asymmetric catalytic cycle as proposed for ABCG5/G8 [38,339,340,341], whereas “symmetric” cycles require the presence of fully conserved “canonical” ATP-binding sites in both NBDs.

Stabilizing salt bridges maintain proper folding and dynamics. At least two salt bridges appear conserved in Cdr1 (Supplementary Figure S1), connecting the elbow helix with the coupling helix (R503 to E576, and R1185 to E1261) (Figure 3D), respectively. However, the salt bridge within ICL1 seems absent in fungal PDR, and a salt bridge at the upper membrane leaflet is found only in the first half of Cdr1 (R456 in ECL1 to E704 in the re-entry helix) (Table 4).

The triple helical bundle (THB) is part of the transmission interface and extremely conserved in Cdr1. Remarkably, the THB is present in diverse ABC transporters, including ABCB1 and LPS extractor, as well as in the antibiotic exporter MacB [332]. This may reflect a universal function in mediating NBD–TMD cross-talk, thus constituting a key element for controlling and driving the conformational switch to drive substrates through the translocation pathway. Moreover, the most highly conserved Y464 residue is essential for ATP consumption, and engages in a salt bridge to stabilize the entire transmission interface in the center of NBD­–elbow–ICL1 cluster (Table 4 and Figure 3C,D). Thus, the THB constitutes a cluster of limited conformational flexibility, taking advantage of Y464 and its salt bridge with E458 in ICL1 and/or between elbow helix R383 and E458 [332] (Table 4 and Figure 3C). As for Cdr1, the proposed THB cluster is present in both N- and C-terminal domains. At the N-terminus, Y584 connects to R503 (elbow helix) and E576 (in the coupling helix), whereas at the C-terminal domain, Y1257 bridges to R1185 (second elbow helix) and E1261 in the second coupling helix (Table 4 and Figure 3D). This finding strongly supports the notion for THB as an essential structure acting at the transmission interface to control the entire transport cycle [332].

The mechanism of intracellular gating in PDR/ABCG2 must be crucial for substrate/inhibitor entry into the transport pathway of the exporter, but as yet is little understood. In human ABCG2, two negative residues are conserved in all ABCG/PDR transporters, represented by E451 (between TMH2 and ICL1) and D477 (beginning of TMH3). Interestingly enough, they are pivotal for drug transport, but do not affect ATP hydrolysis [40] (Table 4 and Figure 3C). Since this region around the transmission interface undergoes dynamic movements during the catalytic cycle, E451 and D477 may not be part of a drug-binding zone but rather provide an entry route and gating functions [332]. In fungal PDRs, both negative residues are conserved (Supplementary Figure S1) and Cdr1 indeed contains the corresponding positions with E570 and E597 in the N-terminal part, and D1255 and E1280 in the C-terminal domain (Table 4 and Figure 3D).

The so-called phenylalanine clamp formed by two F residues is located in the substrate-binding zone of ABCG/PDR transporters. Interestingly, the THM2 in the ABCG/PDR subfamily contains at least 4–5 conserved F residues (Supplementary Figure S1). TMH2 occupies space in the middle of the transmembrane core, where a putative binding zone around the central cavity is present. In human ABCG2, F439 (Figure 3E), located in the middle of TMH2, is implicated in binding both substrates and inhibitors [333]. Remarkably, a new cryo-EM structure of ABCG2 illustrates that the aromatic side chains of both phenylalanine residues could contribute to a binding site [259], and thus play a role as a clamp for both substrate and inhibitor recognition [259,333]. Remarkably, the Cdr1 homology model suggests that the conserved residues in both TMDs (Supplementary Figure S1) are F559 in TMH2 and F1239 in TMH8 (Figure 3F) and equivalent to F439 in ABCG2 (Figure 3E). Hence, conserved phenylalanine in or nearby substrate/inhibitor-binding zones of ABCG/PDR provide a clamping mechanism to trap substrates and/or inhibitors.

Extracellular gating at the membrane interface and subsequent drug release from the outward-facing state is regulated by two conserved motifs, a hydrophobic valve and a flexible lid architecture in the roof [42]. The hydrophobic valve in ABCG/PDR transporters is contributed by two half-molecules, thus generating a physical gate for outward-directed substrate translocation from the central into the upper cavity. The atomic structures of mammalian ABCGs [38,256,259] indeed reveal a unique valve-like motif in the core of the transporter, separating the central cavity from the upper cavity. This conserved “glycine-Φ-Φ” motif plays a critical role as a hydrophobic valve that controls water flow through the transport pathway [42] (Supplementary Figure S1). The glycine adds a flexible kink, whereas two hydrophobic aliphatic leucines build a hydrophobic barrier to prevent water flow or substrate leakage. Human ABCG2 has the “G553-L554-L555” motif, whereas Cdr1 has “G672-F673-V674” and “G1362-V1363-L1364” in the first and second half, respectively (Table 4 and Figure 3G,H). Interestingly, a similar extracellular gating mechanism may also operate in CmABCB1, which possesses a gate at the outer membrane border to regulate substrate translocation [35,295].

A flexible but compact lid is part of the roof architecture formed by a rather large extracellular domain in ABCG/PDR. The roof is another unique motif in the ABCG/PDR subfamily. In human ABCG2, this roof is maintained by an intramolecular C592–C608 disulfide bond that strengthens the compact lid architecture. This lid may establish the second gating mechanism to regulate drug release from the outer cavity [334,335] (Figure 3G). Notably, the covalent C603–C603 inter-molecular link is key for homo-dimer formation in human ABCG2, but not essential for function [334,342]. The last extracellular loop of fungal PDR is slightly larger than the equivalent loop in mammalian ABCG2 (Supplementary Figure S1), where 2–3 conserved cysteine residues are present (Table 4). The overall similarity in the roof architecture also supports the notion of a conserved extracellular gating mechanism in ABCG/PDR transporters (Figure 3G,H).

## 5. Model for a Conserved Catalytic Transport Cycle of ABCG/PDR Transporters

**Figure 4:**
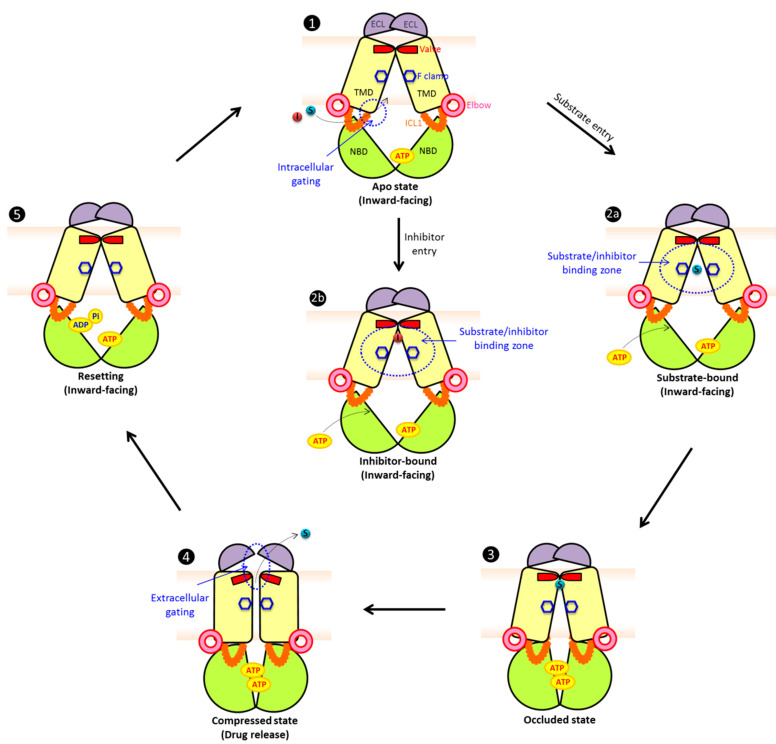
Proposed catalytic cycles of mammalian/fungal type II exporters (ABCG/PDR). In the apo substrate-free state (**1**), the exporter adopts an inward-facing conformation. We propose that NBDs are open with ATP present in at least one NBD or both, which mediates partial NBD dimerization leaving only one accessible ATP-binding region. The intracellular gate(s) at the transmission interface provides access for substrate or inhibitor entry. The aromatic rings at the conserved F clamp form accessible binding sites at the closed transporter valve subtending the closed ECL. Drug substrates (**2a**) or inhibitors (**2b**) can enter through intracellular gate(s), preceding their trapping in distinct binding zones in the central cavity. Binding of ATP at the second binding site or both triggers full NBD dimerization and triggers a first conformational change, setting an occluded state (**3**). The communication between NBD and TMD is regulated via a rigid triple helical bundle (THB) as a key part of the transmission interface. The NBD dimerization compresses the central cavity space to drive substrate movement through the translocation, thus engaging a push and squeeze motion to open the valve. Substrates shift into the upper cavity and are released by the subsequent opening of the ECL lid (**4**). ATP hydrolysis at one NBD site may be enough to reset the catalytic cycle and to convert the transporter molecule into the inward-facing drug-recognizing state (**5**). The structures show NBDs (green), elbow helix (pink), TMDs (yellow), ICL (orange), ECL (purple), phenylalanine clamp (blue hexagon), valve (red), substrates (cyan) and inhibitors (red). For more details and references see the main text.

Mammalian ABCG and fungal PDR share all hallmark domains as well as numerous conserved residues essential for function (Figure 3). Based on these striking similarities, we wish to propose a unified mechanism for the transport cycle of type II ABCG/PDR multidrug transporters (Figure 4). Several studies suggest that more than half of all known ABC transporters including human ABCG5/G8 utilize non-equivalent or deviant NBDs [43,216,259,306,329,343,344]. Interestingly, ABCG2 appears as a perfect homo-dimer molecule, although it may have some asymmetries in the NBD dimers [325,341,345]. Therefore, the “primordial” alternating access model [30,36,324,346,347] forms a rational basis for our model (Figure 4). The ABCG/PDR transporters in the apo drug-free state are in an inward-facing configuration, with the bottom of the NBD dimer connected. We propose that ABCG/PDR subfamilies have asymmetric catalytic cycles, as proposed for human ABCG2 [341]. In this state, only one nucleotide-binding site is occupied by ATP, which could support an “intermediate” NBD dimerization state, with one free site remaining accessible for ATP. The intracellular gate formed by two negative residues at the membrane border of the ICL1 in the transmission interface is open, thus offering a path for substrate/inhibitor entry [40]. The central cavity provides free binding zones to accommodate compounds of variable chemical spaces [157,348]. The aromatic rings in the conserved F clamp establish accessible binding/trapping sites [333]. The hydrophobic valve at the top of the central cavity is almost completely closed and blocks water leakage through the translocation pathway [42]. The lid-forming roof architecture also remains closed in the inward-facing state, with a compact loop structure that limits the space in the upper cavity. Since ABCG/PDR exporters have an uncoupled ATP hydrolysis cycle, the catalytic cycle would still be active even without substrate(s) [33,40,42]. Drug substrates (2a) or inhibitors (2b) can access the central cavity and the translocation pathway through the intracellular gate(s) [40] before getting trapped in the binding zones [157] by the F clamp located in TMH2 in each TMD [333]. This is a critical step which also prevents substrate escape from the central transport pathway. By contrast, the binding of an inhibitor (2b) at the region below the valve, would lock the conformation in the inward–open state and inhibit ATP hydrolysis activity as indicated by cryo-EM particle structures [257]. Whether a compound is an inhibitor or a transport substrate for ABCG/PDR transporters is solely determined by the affinity that sets the on versus off rates, and by the kinetics underlying the interactions in the binding zones [33]. Indeed, biochemistry data also suggest that certain inhibitors inhibit ATPase activity [349,350,351]. Binding of the second ATP molecule functions as a molecular glue that triggers complete NBD dimerization, thus inducing the conformational switch of the TMDs into a substrate-occluded state (3). The mechanical movement at the transmission interface requires the THB as a rigid structure of limited dynamics [332]. Subsequently, the central cavity is compressed, hence creating peristaltic pressure that drives substrates along the central translocation channel through the concomitantly opening valve. Further, the full NBD dimerization pushes the transporter into a compressed state, imposing a squeezing motion on the central cavity that generates pressure critical for opening the valve. The hydrophobic valve also serves as the first barrier for an extracellular gating to ensure unidirectional transport. A retrograde backflow of substrates is therefore prevented by the outward hydrostatic pressure and by the tight valve that would close when resetting the transporter [42]. Accordingly, the space of the upper cavity is then enlarged to accommodate substrates [42]. The limited dynamics and stability of the extracellular roof is supported by a conserved salt bridge between ECL1 and the re-entry helix [42]. The compact lid, which is mainly formed by ECL, then constitutes the second barrier at the extracellular interface. Once the lid has opened, it allows for substrate release into the extracellular space. Finally, ATP hydrolysis at one (or both sites) releases Pi and ADP, thus initiating the reset of the transporter with two NBDs in the original inward–open-facing state [43]. The resulting accessible ATP site also opens the intracellular gate enabling a new cycle of substrate recognition. This catalytic cycle reflects the current knowledge about the catalytic cycles of ABCG2/PDR transporters, whereby biochemical, structural, genetic and mutational data have been integrated.

## 6. Conclusions and Future Perspectives

The increase in atomic structures of ABC transporters, and data from extensive biochemical and genetic experiments, have been propelling the field, since they have yielded novel insights and a better understanding of ABC transporter mechanisms. At the same time, while atomic structures have been invaluable, they have to be interpreted with caution, especially when biochemistry or genetics are not in line with structural data [265] or when biological relevance appears doubtful. Indeed, the painful history of ABC transporter structures [207,265], shows that even higher resolution structures suffer from their static nature that only reflects snapshots of a catalytic cycle. Thus, we need atomic structures reflecting more than a single conformation and possibly many transition states [352], as well as extensive validation by biochemistry and genetics, to validate their biological relevance and define catalytic cycles. Furthermore, there is an unmet need for more interdisciplinary collaborations that also engage alternative structural approaches like NMR [353,354,355,356] as well as complementary biophysical methods [265,357,358,359] to expand our mechanistic views of ABC transporters in all living kingdoms.

The past few years challenge previous notions that a unified transport mechanism exists for MDR exporters from all three major classes, such as ABCB1/MDR1/P-gp [35,293,295,302,303,307,310], ABCC1/MRP1 [298,311,312] and ABCG2/BCRP [256,257,258,259] (Table 3). While early homology modeling attempts of the ABCG/PDR family yielded incorrect folds due to using type I exporter templates rather than exporter type II [360,361,362,363], the use of proper coordinates have now validated the usefulness of modeling for dissecting mechanisms of ABC transport cycles [40,256]. Of note, we have taken a “reverse” approach here, since we exploited human ABCG2 and ABCG5/G8 structures to model evolutionarily conserved fungal PDR transporters. This not only yielded new testable homology models, but also hinted that catalytic cycles may have been conserved at least in orthologous families such as human ABCG and fungal PDR. Of note, four cryo-EM structures of yeast Pdr5 [339] at atomic resolutions from 2.8 to 3.5 Å just emerged [339]. Most remarkably, as we show here for Cdr1, the paradigm yeast Pdr5 efflux pump shows similar transitional movements during the catalytic cycle [339], strongly supporting the proposed catalytic cycle for PDR/ABCG transporters operating as uncoupled peristaltic pumps [339]. *Twist* and *Squeeze* may be used by different types of transporters, and these driving mechanisms may appear related or even similar when looking at it from the mechanics, as either *Twist* or *Squeeze* or a combination of both can result in peristaltic pressure during the switch. While there are many challenges remaining ahead of us, the reversal of clinical MDR phenomena in fungal pathogens in infectious disease settings have regained attention, especially since the catalytic cycle of human ABCG2 likely reflects the mode of action of fungal PDR transporters implicated in anti-infective drug resistance.

## Figures and Tables

**Table 1:** Known ABC transporters in non-pathogenic yeasts.

	Phylogeny	Species	Gene Name	UniProt ID	Length	Substrates	References
Exporter type II	ABCG/PDR	*S. cerevisiae*	*PDR5*	P33302	1511	Drugs, PC, PE, PS, Steroids, Herbicides	[163,164,165,166,167,168,169,170,171,172]
			*PDR10*	P51533	1564	Regulate PDR12 trafficking, Herbicides, Lipids	[173,174,175]
			*PDR11*	P40550	1411	Sterol	[99,176]
			*PDR12*	Q02785	1511	Weak acids, Fluorescein	[177,178]
			*PDR15*	Q04182	1529	Herbicide, Detergent	[174,175,179]
			*PDR18*	P53756	1333	Herbicides, Ethanol, Ergosterol	[180,181,182,183]
			*AUS1*	Q08409	1394	Sterol	[99,158,184]
			*SNQ2*	P32568	1501	Drugs, Steroids, Mutagens, Chemicals	[169,185,186]
		*S. pombe*	*BFR1*	P41820	1530	Brefeldin A, Tributyltin	[187]
Exporter type I	ABCB/MDR	*S. cerevisiae*	ATM1 *	P40416	690	Fe/S proteins	[188,189,190]
			MDL1 *	P33310	695	Peptides	[191,192]
			*STE6*	P12866	1290	a-factor	[193,194]
		*S. pombe*	HMT1 *	Q02592	830	Phytochelatin conjugated Cd2+	[195,196]
			*MAM1*	P78966	1336	M-factor	[195,196]
	ABCC/MRP	*S. cerevisiae*	*YOR1*	P53049	1477	Oligomycin, Reveromycin, Beavericin, Metal ions	[197]
			YCF1 *	P39109	1515	GS-conjug. Cd2+, Metals	[170,198,199,200]
			YBT1 *	P32386	1661	Metal ions, Bile acid, PC	[201,202]
			VMR1 *	P38735	1592	Drugs, Metal ions	[203]
			BPT1 *	P14772	1559	Metal ions, Bile acid, GS-conjugates	[204]
		*S. pombe*	*PMD1*	P36619	1362	Drugs	[205]

* All transporters located in the plasma membrane, except for Atm1, Mlt1 and Ybt1, Vmr1, Ycf1 and Hmt1, residing in the mitochondrial and vacuolar membrane. GS: glutathione, PC: phosphatidylcholine, PE: phosphatidylethanolamine, PS: phosphatidylserine.

**Table 2:** ABC efflux transporters mediating MDR in pathogenic fungi.

	Phylogeny	Species	Gene	UniProt ID	Length	Substrates	References
Exporter type II	ABCG/PDR	*C. albicans*	*CDR1*	P43071	1501	Drugs, PC, PE, PS, Steroids	[217,260,263,267,268,269,270]
			*CDR2*	P78595	1499	Drugs, PC, PE, PS, Steroids	[218,260,271]
			*CDR3*	O42690	1501	Drugs, PC, PE, PS, Steroids	[260,272]
			*CDR6/ROA1*	A0A1D8PCM6	1274	Azole, Membrane fluidity	[273]
			*CDR11*	A0A1D8PK15	1512	Drugs, Fosmanogepix	[274]
		*C. glabrata*	*CDR1*	Q6FK23	1499	Drugs	[275,276]
			*PDH1*	O74208	1542	Drugs	[277,278]
			*SNQ2*	Q6FQN3	1507	Drugs	[279]
			*AUS1*	Q6FUR1	1398	Sterol	[280,281]
		*C. auris*	*CDR1*	PIS57142	1508	Drugs	[236,237,239]
		*C. dubliniensis*	*CDR1*	Q8NJ55	1501	Drugs	[282]
			*CDR2*	Q8NJ54	1500	Drugs	[282]
		*C. tropicalis*	*CDR1*	EER33788	1498	Drugs	[283,284,285]
		*C. neoformans*	*AFR1*	Q8X0Z3	1543	Drugs	[286]
Exporter type I	ABCB/MDR	*C. albicans*	*HST6*	A0A1D8PKI5	1323	A-factor	[287,288]
		*C. glabrata*	ATM1 *	Q6FIK3	727	Fe/S	[289]
		*C. neoformans*	*MDR1*	O43140	1408	Drugs	[290]
			ATM1 *	P0CL92	734	Fe/S	[290]
	ABCC/MRP	*C. albicans*	*YOR1*	AOW27977	1488	Oligomycin, Beauvericin	[291,292]
			MLT1 *	Q9UW87	1606	PC, Ni(II)	[180]

* All transporters located in the plasma membrane, except for Atm1 and Mlt1, residing in the mitochondrial and vacuolar membrane, respectively. GS: glutathione, PC: phosphatidylcholine, PE: phosphatidylethanolamine, PS: phosphatidylserine.

**Table 3:** Atomic structures of eukaryotic MDR ABC transporters.

Subfamily	PDB ID	Function	References
ABCB1 (P-gp)	4F4C, 4M1M, 4M2S, 4M2T, 4Q9H, 4Q9J, 4Q9K, 4Q9L, 4XWK, 5KPF, 6KPI, 5KPJ, 5KO2, 5KOY, 6C0V, 6GDI, 6Q81, 6QEX, 6QEE, 6FN4, 6FN1, 3G5U, 3G60, 3G61	Multidrug export, detoxification	[255,296,302,303,304,305,306,308,309,310]
ABCC1 (MRP1)	5UJA, 5UJ9, 6BHU, 6UY0	Multidrug, leukotriene and sphingolipid export, detoxification	[298,312]
ABCG2 (BCRP)	5NJG, 5NJ3, 6ETI, 6FEQ, 6HIJ, 6HCO, 6HBU, 6HZM, 6VXH, 6VXI, 6VXJ	Multidrug export, detoxification and urate transport	[256,257,258,259]

**Table 4:** The human ABCG2 and fungal Cdr1 multidrug exporters share conserved motifs.

Location	Conserved Motif	Functional Role	Human ABCG2	*Candida albicans* Cdr1	
				First Half	Second Half
NBD	Walker A	ATP hydrolysis (phosphate binding) *	G79–S88	G187–T195	G895–T903
	Q-loop	TMD–NBD communication	Q126	E238 **	Q942
	Hot spot	Triple helical bundle	L134–A149	L246–P261	S950–S965
	Signature	NBD dimerization and phosphate binding	V186–R193	V303–R310	V996–R1008
	Pro loop	NBD dimerization	P204	?	P1019
	Walker B	ATP hydrolysis	I206–E211	I323–N238	L1021–E1027
	D-loop	NBD dimerization	L216–D217	L333–D334	L1032–D1033
	H-loop	ATP hydrolysis	H243	Y361 **	H1059
Elbow helix	Conserved R	Salt bridge, THB	R383	R503	R1185
ECL1	Conserved R	Salt bridge	R426	R456	?
TMH2	Conserved F	Clamping	F439	F559	F1239
ICL1	Conserved E (1)	Salt bridge and intracellular gating	E451	E570	D1255
	Conserved E (2)	Salt bridge	E458	E576	E1261
	Conserved Y	Salt bridge, THB	Y464	Y584	Y1257
TMH3	Conserved D/E	Intracellular gating	D477	E597	E1280
Valve	Conserved hydrophobic	Valve	G553–L555	G672–V674	G1362–L1364
Re-entry helix	Conserved P	Kinked helix	P574	P692	P1382
	Conserved E	Salt bridge	E585	E704	?
ECL3	Conserved C (1)	Intra/intermolecular disulfide bond	C592	C712	C1418
	conserved C (2)	Intra/intermolecular disulfide bond	C603	?	C1441
	conserved C (3)	Intra/intermolecular disulfide bond	C608	C732	C1444

* Some PDRs may consume GTP as well [186,331]. ** Substitution with another residue.

## Data Availability

The authors confirm that all data are fully available without restrictions. All relevant data are provided in the manuscript and in the Supplementary Materials.

## Supplementary Materials

The following are available online at https://www.mdpi.com/article/10.3390/ijms22094806/s1. Figure S1. Amino acid sequence alignment of fungal ABC transporters (PDR subfamily) and mammalian ABCG subfamily.

## Data and Analysis

Figures and phylogenetic analyses were generated by BioEdit, ClustalX2 and MEGA-X. The homology model was generated using SWISS-MODEL using a Cdr1 sequence from UniProt (accession no. P43071), using the human ABCG2 as a template (PDB ID 6VXF). The molecular visualizations were performed using PyMOL, v1.8.4.
