# Supplementary material for: Multidrug Resistance in Mammals and Fungi—From MDR to PDR: A Rocky Road from Atomic Structures to Transport Mechanisms
Source: Int J Mol Sci. 2021 Apr 30;22(9):4806. doi: 10.3390/ijms22094806 (PMC8124828; doi:10.3390/ijms22094806)
Supplement: Supplementary file 1 [file ijms-22-04806-s001.zip › IJMS-1178190-Supplementary.pdf]

Supplementary Information

Multidrug Resistance in Mammals and Fungi—From MDR to PDR: A Rocky Road from Atomic Structures to Transport Mechanisms

Narakorn Khunweeraphong and Karl Kuchler *

Center for Medical Biochemistry, Max Perutz Labs Vienna, Campus Vienna Biocenter, Medical University of Vienna, Dr. Bohr-Gasse 9/2, A-1030 Vienna, Austria; narakorn.khunweeraphong@meduniwien.ac.at
* Correspondence: karl.kuchler@meduniwien.ac.at; Tel.: +43-1-4277-61807; Fax: +43-1-4277-9618

Keywords: yeast; multidrug transporter; anticancer; antifungal resistance; ABC transporters; mechanism

Supplementary Figures and Legends

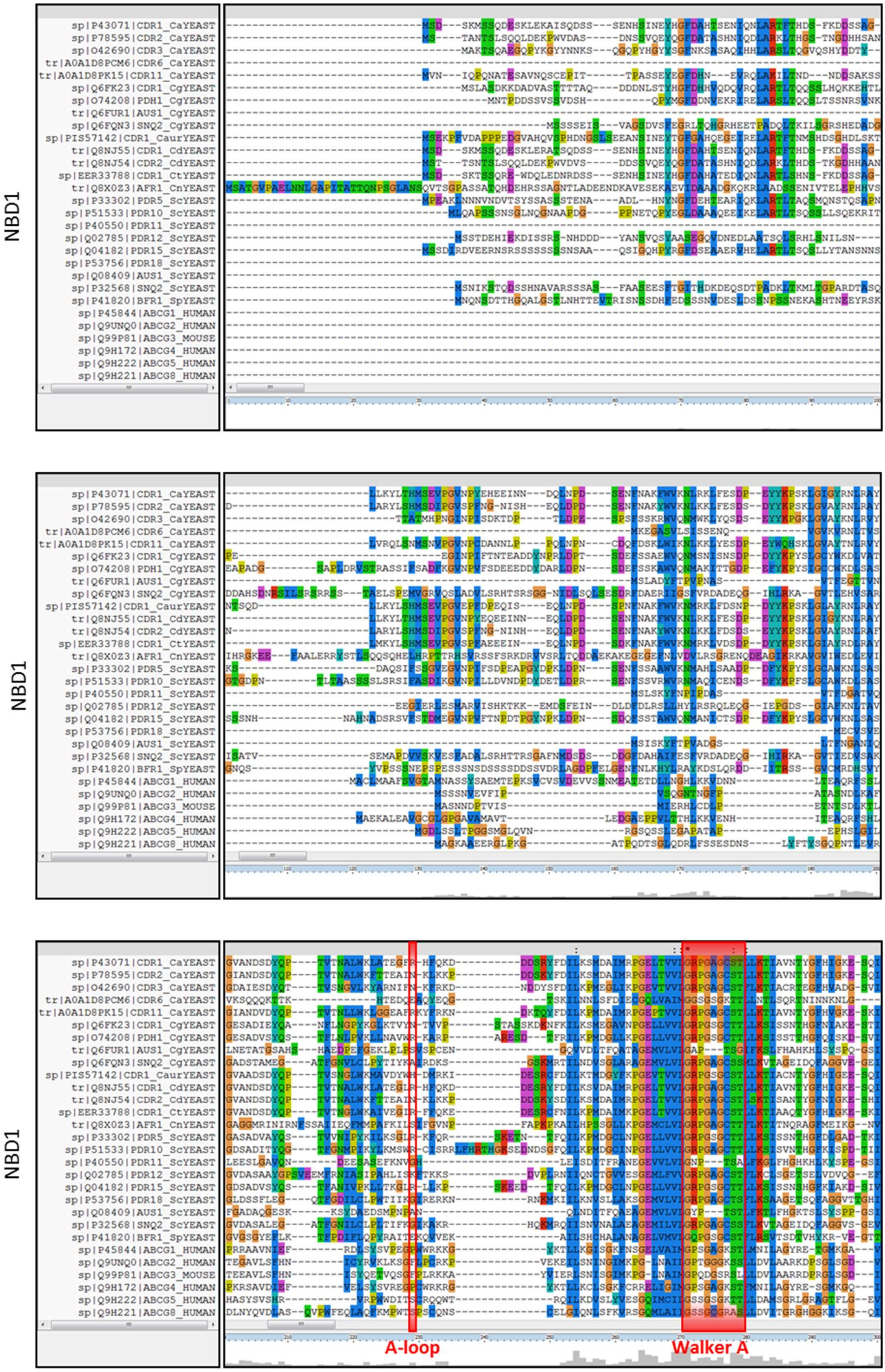

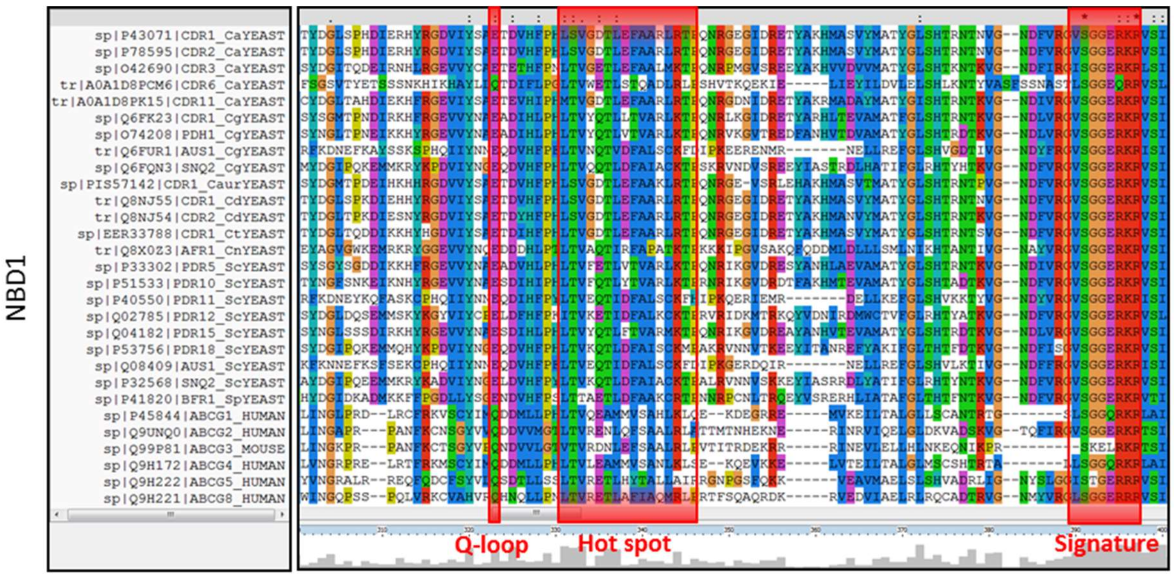
NBD1
Q-loop
Hot spot
Signature

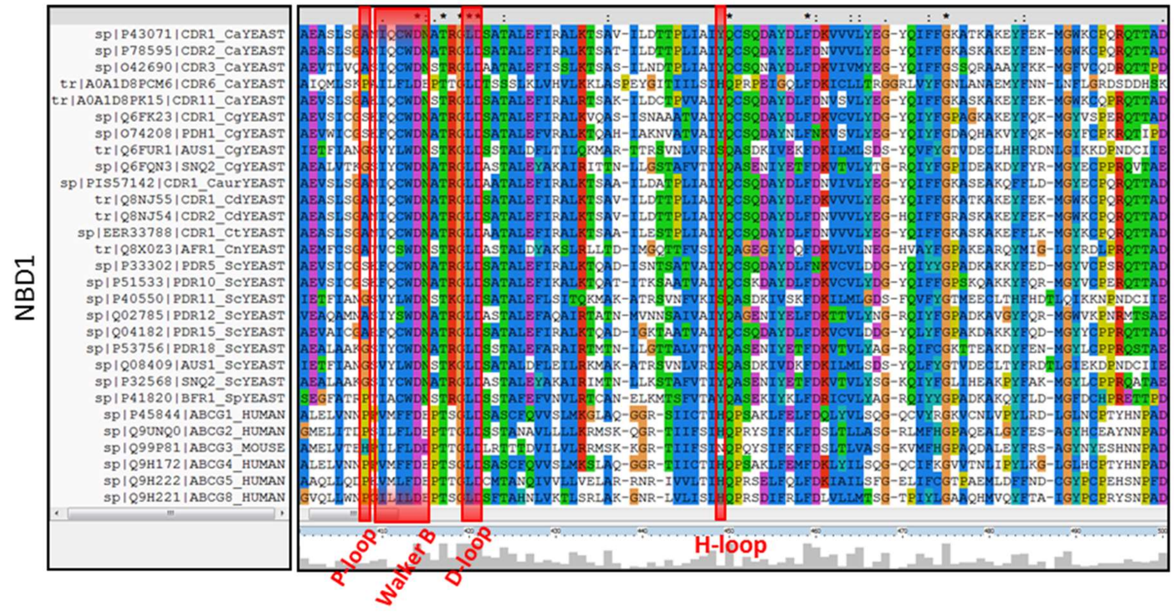
NBD1
P-loop
Walker B
D-loop
H-loop

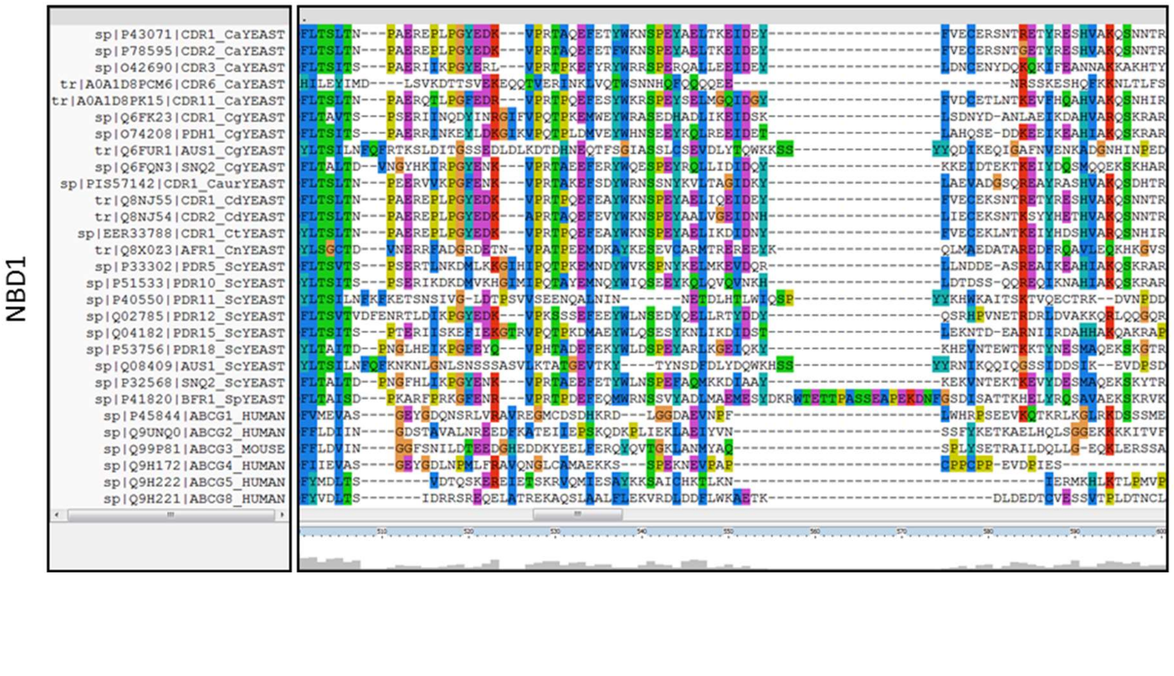
NBD1

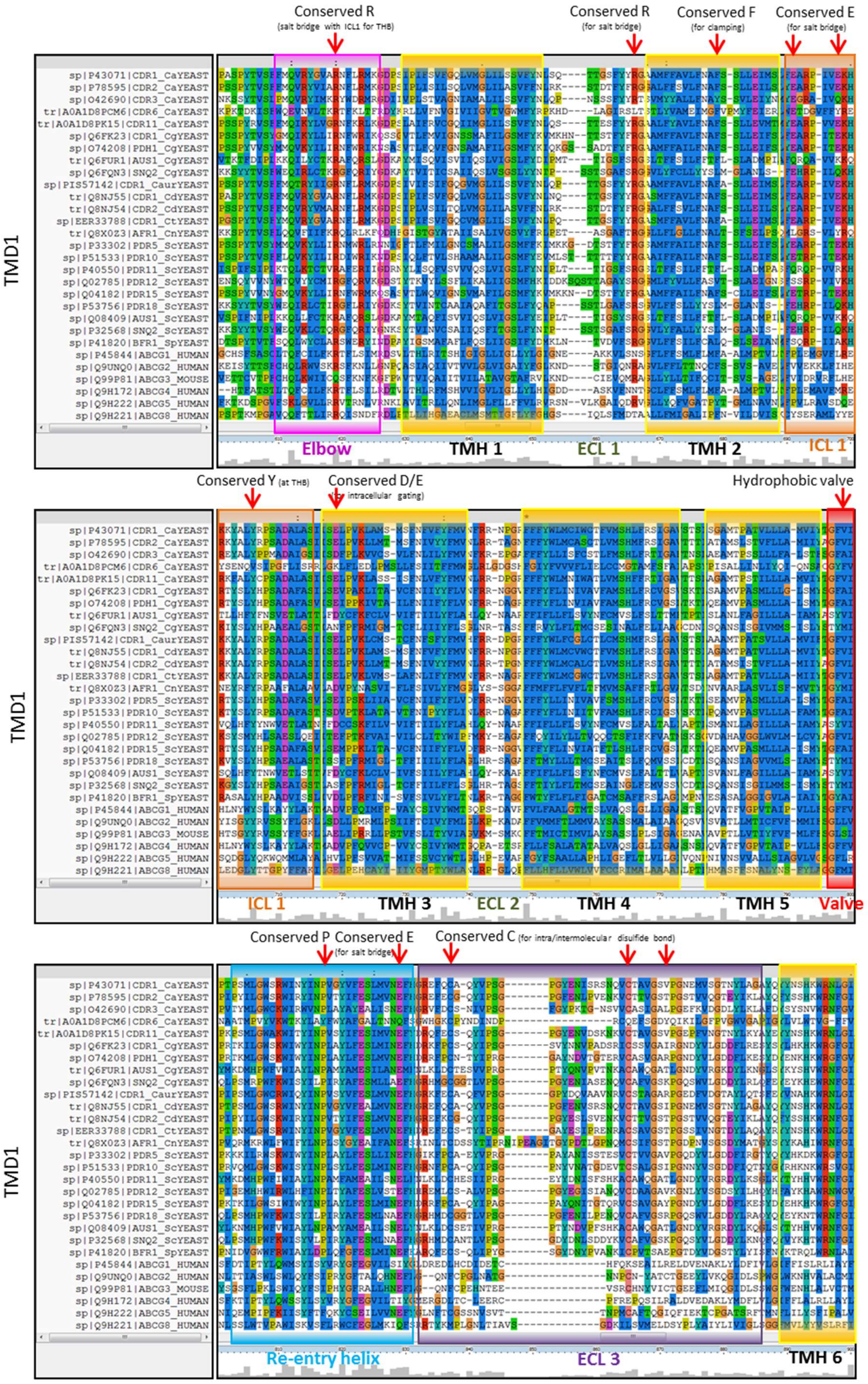
Conserved R
(salt bridge with ICL1 for THB)
Conserved R
(for salt bridge)
Conserved F
(for clamping)
Conserved E
(for salt bridge)
TMD1
Elbow
TMH 1
ECL 1
TMH 2
ICL 1
Conserved Y (at THB)
Conserved D/E
Hydrophobic valve
TMD1
ICL 1
TMH 3
ECL 2
TMH 4
TMH 5
Valve
Conserved P
Conserved E
(for salt bridge)
Conserved C (for intra/intermolecular disulfide bond)
TMD1
Re-entry helix
ECL 3
TMH 6

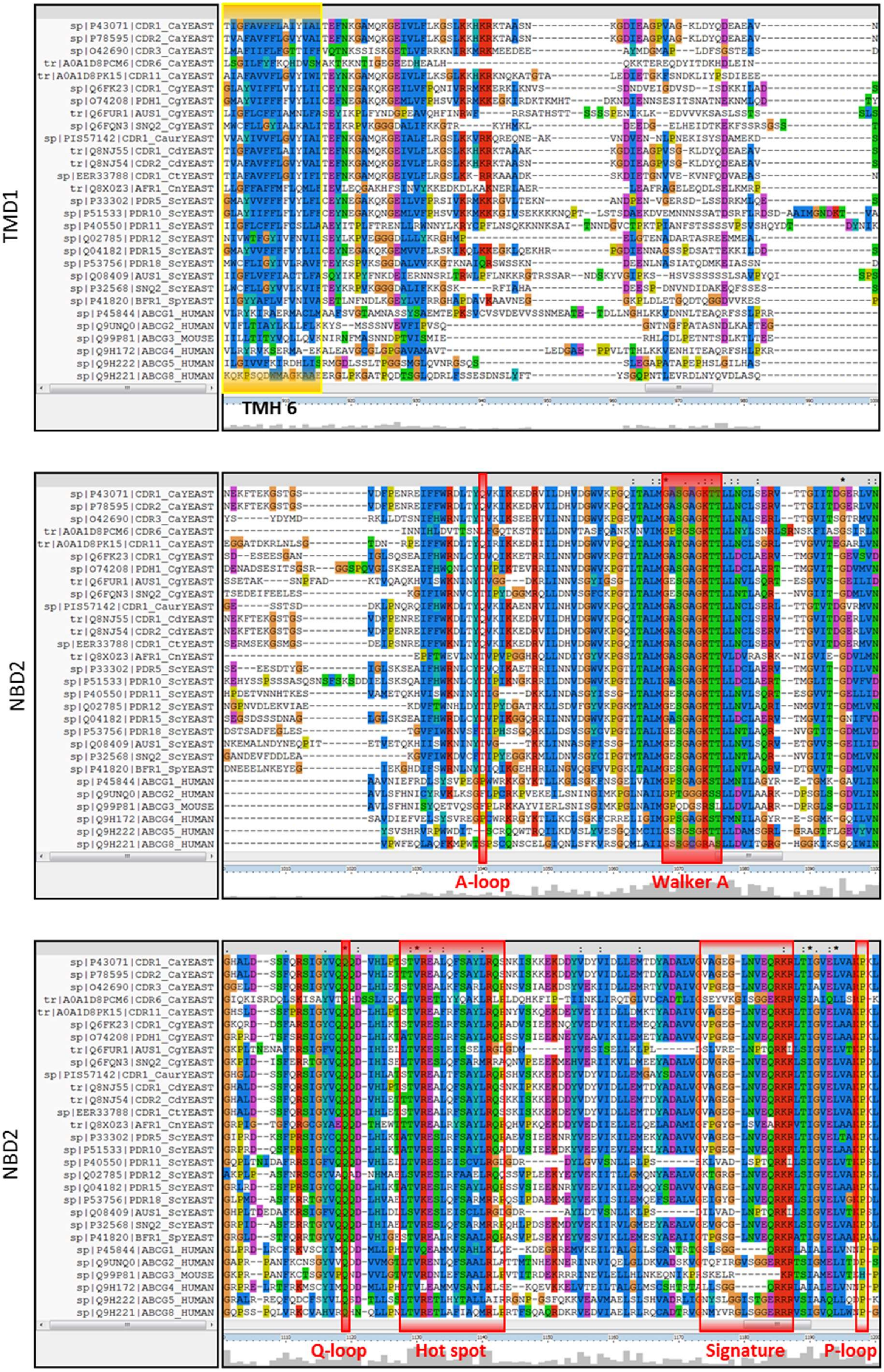
TMD1
TMH 6
NBD2
A-loop
Walker A
NBD2
Q-loop
Hot spot
Signature
P-loop

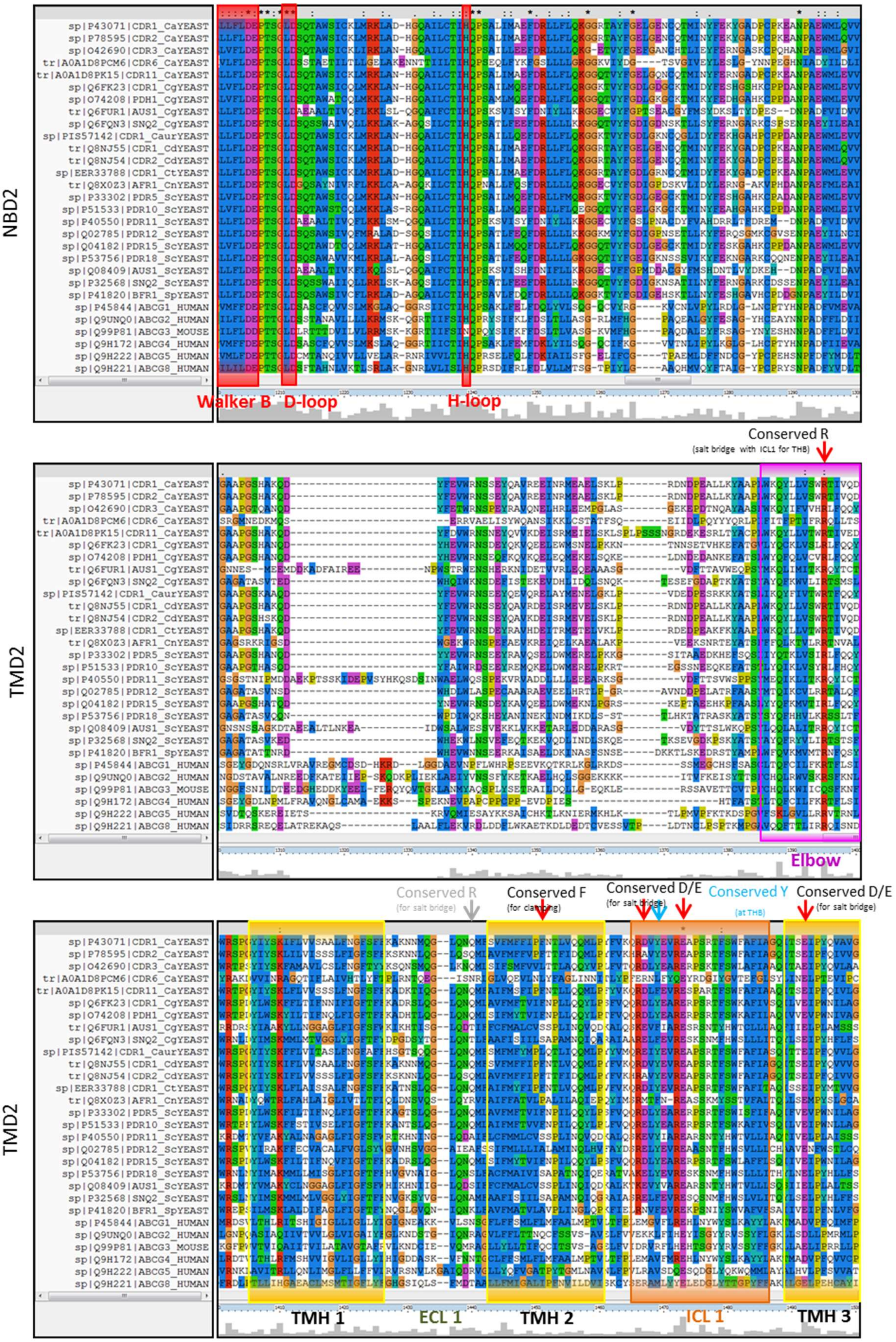
NBD2
Walker B
D-loop
H-loop
Conserved R
(salt bridge with ICL1 for THB)
TMD2
Elbow
Conserved R
(for salt bridge)
Conserved F
(for clamping)
Conserved D/E
(for salt bridge)
Conserved Y
(at THB)
Conserved D/E
(for salt bridge)
TMH 1
ECL 1
TMH 2
ICL 1
TMH 3

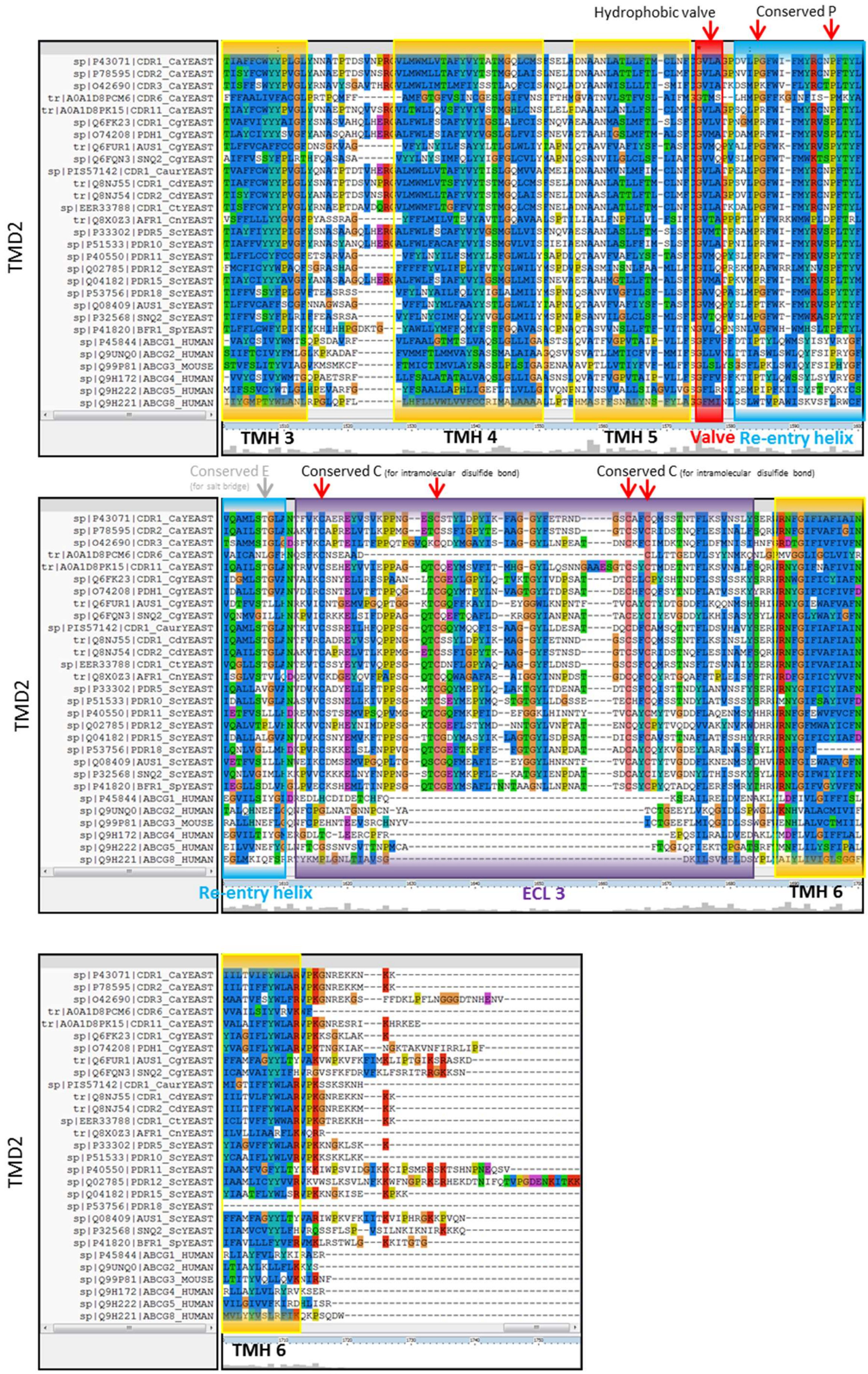
Hydrophobic valve
Conserved P
TMD2
TMH 3
TMH 4
TMH 5
Valve
Re-entry helix
Conserved E
(for salt bridge)
Conserved C (for intramolecular disulfide bond)
Conserved C (for intramolecular disulfide bond)
TMD2
Re-entry helix
ECL 3
TMH 6
TMD2
TMH 6

Supplementary Figure S1: Amino acid sequence alignment of fungal ABC transporters (PDR subfamily) and mammalian ABCG subfamily.

The multiple sequence alignments (MSA) were conducted using BioEdit and ClustalX2. Conserved residues are highlighted with the conservation scale as the height of gray bars at the bottom. Conserved motifs in the NBDs are highlighted in the red boxes, including A-loop, Walker A, Q-loop, hot spot helix, Signature loop, Pro-loop, Walker B, D-loop and H-loop, respectively. The putative conserved motifs in the TMDs are highlighted in color boxes, the elbow helix (pink), TMH1-TMH6 (yellow), ICL (orange), re-entry helix (blue) and ECL (violet), respectively. Ca: *Candida albicans*, Cg: *Candida glabrata*, Caur: *Candida auris*, Cd: *Candida dubliniensis*, Ct: *Candida tropicalis*, Cn: *Cryptococcus neoformans*, Sc: *Saccharomyces cerevisiae*, Sp: *Schizosaccharomyces pombe*.
